# The Role of BAR Domain Proteins in the Regulation of Membrane Dynamics

**Published:** 2016

**Authors:** T.B. Stanishneva-Konovalova, N.I. Derkacheva, S.V. Polevova, O.S. Sokolova

**Affiliations:** Lomonosov Moscow State University, Faculty of Biology, Leninskie Gory 1, Bld 12, Moscow, 119234 , Russia; A.I. Evdokimov Moscow State University of Medicine and Dentistry, Department of Biochemistry, Delegatskaya Str. 20, Bld 1, Moscow, 127473, Russia

**Keywords:** BAR domains, lipid membranes, membrane dynamics

## Abstract

Many cellular processes are associated with membrane remodeling. The BAR domain protein family plays a key role in the formation and detection of local membrane curvatures and in attracting other proteins, including the regulators of actin dynamics. Based on their structural and phylogenetic properties, BAR domains are divided into several groups which affect membrane in various ways and perform different functions in cells. However, recent studies have uncovered evidence of functional differences even within the same group. This review discusses the principles underlying the interactions of different groups of BAR domains, and their individual representatives ,with membranes.

## INTRODUCTION

**Fig. 1:**
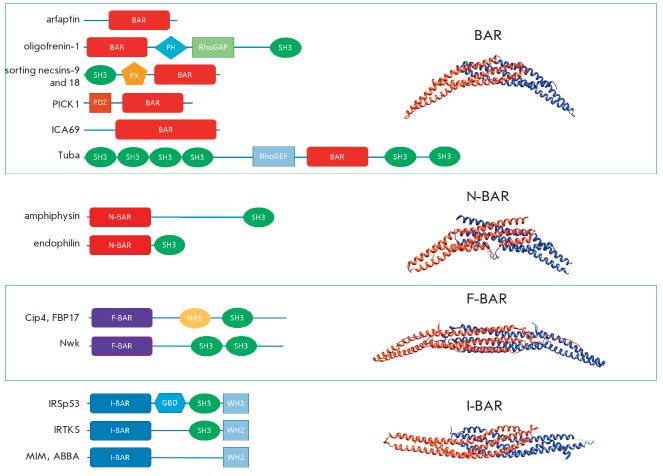
The domain structures of BAR family members (left side) and the structures of BAR domain dimers (right side).

During cell movement, the coordinated processes of actin polymerization and the interaction between actin filaments and the cellular membrane push the active cell edge forward and result in filopodia formation. These processes are coordinated by actin-binding proteins. Disruptions in the function of actin-binding proteins that infringe on cell motility are a distinct feature of neoblasts. BAR family proteins act as connecting links between actin dynamics and membrane rearrangements in all eukaryotes. BAR domains were originally defined as the conserved regions of the animal proteins BIN and amphiphysin, as well as the yeast Rvs161 and Rvs167 proteins [1]. Along with the BAR domain, the proteins belonging to this family contain other domains that are required to ensure that they bind to specific proteins and lipids, which determines their function and arrangement in a cell [2] (*Fig. 1*). The preferential binding of BAR domains to curved membrane regions makes it possible to attract target proteins to membrane rearrangement sites.

There are different ways through which BAR domain proteins can affect actin polymerization. In some cases, they activate the actin nucleation factors WASP (Wiskott-Aldrich Syndrome Protein) and WAVE (Wiskott-Aldrich Verprolin Protein) [3], while other BAR domain proteins interact with Rho GTPases [4]. Most BAR domains attract the proteins specific to a certain cellular process to the membrane thanks to SH3 domains, which can interact with a number of proteins that contain proline-rich sequences [5, 6]. This fact raises the question of which factors determine the specificity of attracting certain proteins. According to the existing hypothesis, partner proteins recognize the spatial arrangement of SH3 domains rather than individual SH3 domains [7] (see text below).

In addition to attracting partner proteins, SH3 domains often function as regulators of the BAR domain activity [8, 9]. Binding of SH3 to the BAR domain typically transforms the structure into an autoinhibited state; this state can be activated only via interaction with an activator protein [9]. In the F-BAR protein Nervous wreck (Nwk), binding of the SH3 domain to F-BAR does not block its membrane-binding ability but only increases the amount of phosphatidylinositol-4,5-bisphosphate (PI(4,5)P_2_) required for the binding [10].

The PICK protein, whose functions have to do with the internalization and exposure of AMPA receptors to the cell surface, provides an interesting example of BAR domain activity regulation [11]. PICK is inhibited by another BAR domain protein, ICA69 [12]. It remains unclear whether this is caused by the formation of a heterodimer from the BAR domains ICA69 and PICK or by the co-oligomerization of their homodimers. The second version seems more plausible, taking into account the stability of the dimers of BAR domain proteins and the potential involvement of the C-terminal region of ICA69 in the interaction.

According to the Uniprot database, the BAR family currently includes more than 220 proteins [13]; crystal structures have been obtained only for 25% of them [14]. The overarching feature of all BAR domains is that they form dimers with the positively charged surface that binds to negatively charged lipid membranes [15, 16]. BAR domains can be classified into several groups based on their structural and phylogenetic properties: classical BAR/N-BAR, F-BAR, and I-BAR (*Fig. 1*) [17].

**Classical BAR domains and N-BAR domains**

A classical BAR domain is a dimer where each monomer consists of three bent antiparallel α-helices [15]. The classical BAR and N-BAR dimers have a crescent shape and bind to the membrane by their concave surface. Most proteins containing the classical BAR domains are present in mammalian nerve cells, where they are involved in the formation of synaptic contacts and in the processes related to signal transduction [18].

Amphiphysins are among the best-studied BAR domain proteins; their functions are associated with neuronal endocytosis [19]. Mammals carry two genes encoding amphiphysins. The amphiphysin II isoform, as well as drosophila amphiphysin, is expressed in muscle cells instead of neurons; it is involved in the formation and stabilization of T-tubules [20, 21]. Mutations in human amphiphysin II/BIN1 cause a hereditary neuromuscular disease known as centronuclear or myotubular neuropathy [22]. The N-terminal BAR domain is the only conserved region of different amphiphysins.

The crystal structure of the BAR domain of drosophila amphiphysin was obtained in 2004, and a prediction was made that similar domains can be found in many protein groups [15]. Based on their structural similarity, the earlier deciphered structure of the C-terminal domain of arfaptin [23] and the endophilin structure deciphered later [24] were classified as belonging to the BAR domain family. By that time, the significant role of endophilin in endocytosis and its interaction with amphiphysin and dynamin had already been reported in a number of studies [25, 26].

**Fig. 2:**
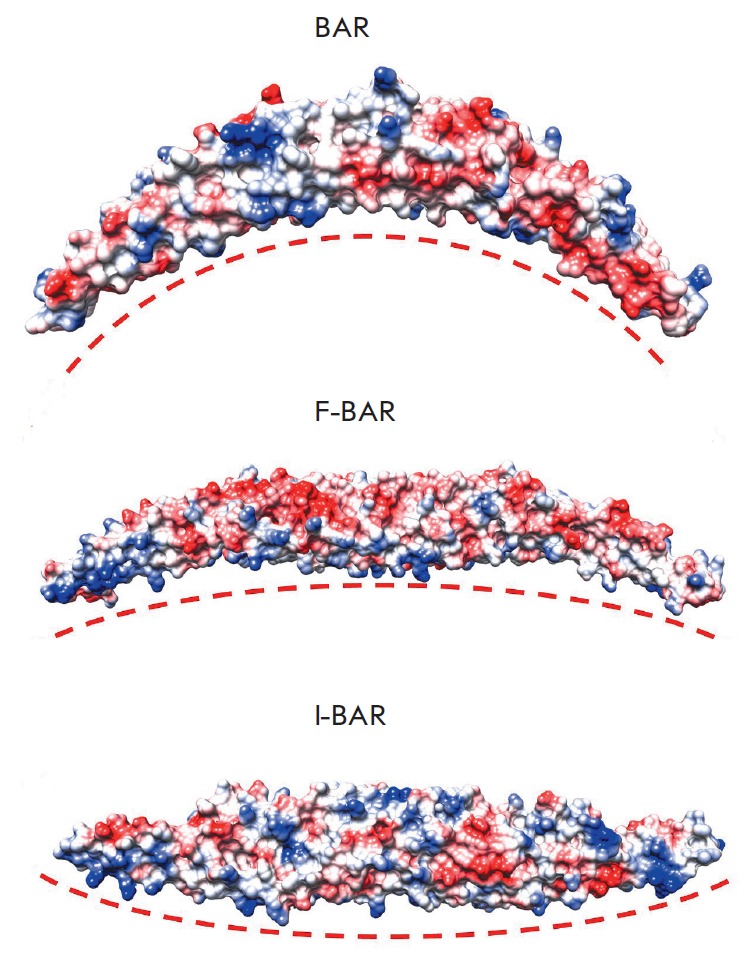
Membrane deformation by BAR domains. The electrostatic surface potentials of the domains are shown with blue as positive and red as negative; the membrane surface is depicted as a red dotted line.

According to X-ray diffraction analysis data, clusters of positively charged amino acid residues (*Fig. 2*) reside at the ends of the BAR domain, between the α-helices 2 and 3 and on its concave surface. Mutations in them reduce the ability of the BAR domain to bind to the membrane and modify liposomes *in vitro*. It has also been shown that the 26 a.a.r. N-terminal sequence of amphiphysin has an unordered structure in the solution but folds into an amphipathic helix (AH) when interacting with lipids. The insertion of an AH helps the BAR domain generate the membrane curvature [27]. AH was subsequently found in many (but not all) BAR domain proteins.

**I-BAR domains**

The I-BAR domain was first determined as a homologous N-terminal domain of mammalian IRSp53 and MIM proteins and called the IM domain (IRSp53/MIM) [28]. Later, due to its structural similarity to the BAR domains, this domain became known as I-BAR (Inverse BAR) [29]. I-BAR domain proteins are present both in higher and lower eukaryotes but have not been found in yeasts.

Similarly to the classical BAR domains, I-BAR domains consist of three α-helices and form dimers; many of these dimers bind to liposomes and modify their curvature in *in vitro *experiments [30-32]. The I-BAR dimer is less curved than the classical BAR (*Fig. 1*). Clusters of positively charged amino acids that are responsible for the binding to negatively charged lipids in the membrane reside on their convex, rather than on the concave, surface (*Fig. 2*) and cause membrane curvature in the opposite direction as compared to BAR’s action [28, 33].

The gene encoding IRSp53 is actively expressed in various mammalian cells and tissues, especially in neurons. IRSp53 knockout mice showed impaired learning skills and memory [34]. IRSp53 contains a CRIB motif that binds to GTPase Cdc42 and an SH3 domain that interacts with WAVE. When bound into a complex with Cdc42 and the Eps8 protein, it can induce filopodia formation [35], while in complex with WAVE it causes lamellipodia formation [36]. IRSp53 is regulated by phosphorylation of two threonine residues, which results in binding of protein 14-3-3 to it and subsequent inactivation [37]. Smaller amounts of IRTKS, the closest homologue of IRSp53, were found in the brain; it was also detected in the bladder, liver, testes, heart, and lungs. Unlike IRSp53, IRTKS does not bind to Cdc42 and its expression in cells causes the formation of clusters of short actin filaments but not filopodia; however, the specific biological functions of IRTKS have not been elucidated yet [38]. MIM (Missing-In-Metastasis) was given its name due to the fact that its expression was reduced in some metastasizing cell lines [39]; however, the more recent studies have demonstrated that its expression can also be elevated in other metastasizing cell lines [40]. MIM is actively expressed in the heart, skeletal muscles, and the central nervous system during ontogenesis. Overexpression of MIM in mammalian cell lines leads to the disappearance of actin stress fibers and emergence of multiple small protrusions on the cell surface [41]. The activity of MIM, identically to that of IRSp53, is regulated by the phosphorylation of the residue in the central portion of the protein (outside the I-BAR domain) [42]. MIM was reported to be involved in cilia formation [43]; however, its accurate role in animal development and physiology remains unclear. The ABBA protein, the closest homologue of MIM, is expressed in glial cells of the murine central nervous system but is absent in neurons. In the glial cell line C6- R, ABBA resides within cortical actin; its knockdown results in defects in lamellipodia formation [44].

The atomic structure of the I-BAR domain of the Pinkbar (Planar Intestinal- and Kidney-specific BAR) protein was deciphered in 2011: the structure is characterized by an almost zero curvature [45]. This protein is expressed in epithelial intestinal and renal cells and partakes in membrane structuration in the intercellular contact zone. The I-BAR domain of the Pinkbar protein, unlike the other known domains, can form flattened membrane regions and aggregate into stable flat oligomers both on the lipid membrane and in solution [29, 45]. The results of domain oligomerization include membrane deformation and clustering of charged lipids PI(4,5)P2 in the membrane. I-BAR domains have a higher electrostatic potential compared to the classical BAR domains and can form PI(4,5)P_2_ clusters at the micron scale [33].

**F-BAR domains**

Another broad group of BAR domain proteins contains the F-BAR domain (Fes/CIP4 homology-BAR). F-BAR proteins were found in most eukaryotes except for plants; they are considered to be the key regulators of cellular membrane curvature [46]. Most of the known F-BAR domain proteins are involved in clathrin-mediated or caveolin-dependent endocytosis. Many of them also partake in the formation of filopodia and lamellipodia: filopodia are required for the formation of axons [47], while lamellipodia inhibit this process [48]. Both these structures can ensure the migration of normal cells and be involved in the dissemination of metastasizing cells [49]. Cell division that also involves F-BAR domain proteins is another crucial process the disruption of which triggers tumor formation. Diseases associated with an altered expression level or mutations in the genes encoding proteins belonging to this family include developmental disorders, neurological and autoinflammatory diseases, invasive tumors, cardiac hypertrophy, carbohydrate metabolism disorder, and renal failure, thus making F-BAR domain proteins a potential therapeutic target [50].

The F-BAR domain was first discovered in the CIP4 protein (CDC42-Interacting Protein 4) [51]. The conserved N-terminal region (60 a.a.r.) of the CIP4 and FES proteins became known as FCH (FES/ CIP4 Homology). It resides next to a domain whose structure is similar to that of the BAR domain and forms a functional unit with it (F-BAR). An analysis of the crystal structures of the F-BAR domains in mammalian FBP17 and CIP4 proteins showed that the shape of F-BAR domains is less curved and more elongated compared to that of classical BAR domains [16] (*Fig. 1*). They consist of five α-helices: the short N-terminal helix, three long and one short C-terminal helices, followed by a short sequence responsible for homodimerization. The surfaces with which monomers interact with each other mostly contain hydrophobic amino acid residues and several charged ones (*Fig. 2*). Mutations in the conserved positively charged amino acid residues on the concave side of F-BAR dimers reduce the ability of proteins to bind to the membrane and modify liposomes *in vitro *[16, 52].

Recent studies demonstrate that some F-BAR domains selectively bind to phosphoinositides [53, 54]. Thus, the yeast protein Rgd1p that activates Rho3 and Rho4 GTPases [55] was found to have a phosphoinositide-binding site that the other F-BAR domain yeast proteins Bzz1p and Hof1p do not have [56]. *In vitro *experiments have demonstrated that Rgd1p preferentially binds to liposomes containing PI(4,5)P2. Deciphering of the crystal structure of the complex between Rgd1p and myo-inositol- 1,2,3,4,5,6-hexakisphosphate (Ins P6), which acts as an analogue of the phosphoinositide lipid head, made it possible to identify which amino acid residues the phosphoinositide-binding site consists of.

The CIP4, FBP17, and FCHo2 proteins also exhibit specificity to phosphoinositides and contain a corresponding binding site [16, 52, 57]. The same site was revealed in human protein Gmip [58], which activates RhoA GTPase and plays a crucial role in cortical actin rearrangement during early mitosis [59] and in neuronal migration [60]. In both processes, phosphoinositides are important regulators. Hence, the specificity of the binding of some F-BAR domains to lipids enables the attraction of F-BAR domain proteins to certain membrane regions. Furthermore, binding of F-BAR domains limits the diffusion of lipids and, therefore, transmembrane proteins, which may be of crucial importance for the spatial arrangement of proteins in a specific cellular process [54, 61].

**The interaction between BAR domain proteins and the membrane**

The main functions of BAR domains include the **generation **of membrane curvature, its **propagation**, **stabilization, **and/or **sensing, **followed by the recruitment of cytosolic protein factors to a specific site in the cell [17]. Generation of the curvature and its propagation are coupled processes: local deformations of the membrane caused by one dimer facilitate the binding of other dimers.

The initial stages of **generation **of the membrane curvature take place due to the electrostatic binding of the BAR domain to the membrane and, in some cases, due to the incorporation of an N-terminal AH into the membrane [62]. Binding is based on the interaction between positively charged amino acids and negatively charged lipids; as mentioned above, some BAR domains preferentially bind to phosphoinositides [56]. The incorporation of AHs into one monolayer facilitates curvature generation due to the asymmetry emerging in the bilayer structure [63]. It was also demonstrated that AHs in some BAR proteins play a key role in the fragmenting of small liposomes [64]. However, the existing experimental data on the binding of BAR domains that carry no AH to membranes [65, 66] do not allow one to unambiguously answer the question about the role of AHs in the generation of membrane curvature.

Curvature **propagation **requires an interaction between many BAR domains. The structure formed by them on the membrane surface is known as a scaffold. All BAR domain proteins are believed to be capable of scaffold formation; the scaffold structure largely determines the result of the effect on the membrane. In its turn, the scaffold structure depends on the protein concentration and membrane tension. It was shown by coarse-grained molecular dynamics simulation that when present at low concentrations, N-BAR domains aggregate on flat membranes and liposomes to form a filamentary structure and networks; after a 20% surface density is achieved, they start forming a membrane protrusion [67]. N-BAR protein endophilin, whose functions are related to endocytosis, can induce tubule formation on the giant liposome at ~5% density and low surface tension (ST). A high protein density is required for tubules to be formed at high ST values; tubule formation is completely inhibited at ST > 0.25 mN/m. The effect of ST on scaffold assembly is caused by the fact that the binding of dimers through their terminal regions is mediated by local membrane deformations, which are impeded by high ST values. This fact suggests that reduced ST can trigger the mechanism of activation of rapid endocytosis [68].

**The sensitivity of BAR domains to membrane curvature**

The investigation of the fluorescence intensity of the proteins on the membrane tubules formed by giant liposomes has shown that BAR domains can act as detectors of membrane curvature: the density of arrangement of the BAR domains bound to membrane tubules is several dozen or even hundreds of times higher than that of the BAR domains residing on a flat membrane. All the tested BAR domain proteins amphiphysin [69], endophilin [70], BIN1 [71], syndapin [65], and IRSp53 [66] have been shown to exhibit preferential binding to membrane tubules. In order to explain why BAR domains with a similar structure have different effects on membranes, let’s discuss the ways in which a number of BAR domains are arranged on the membrane.

**Fig. 3:**
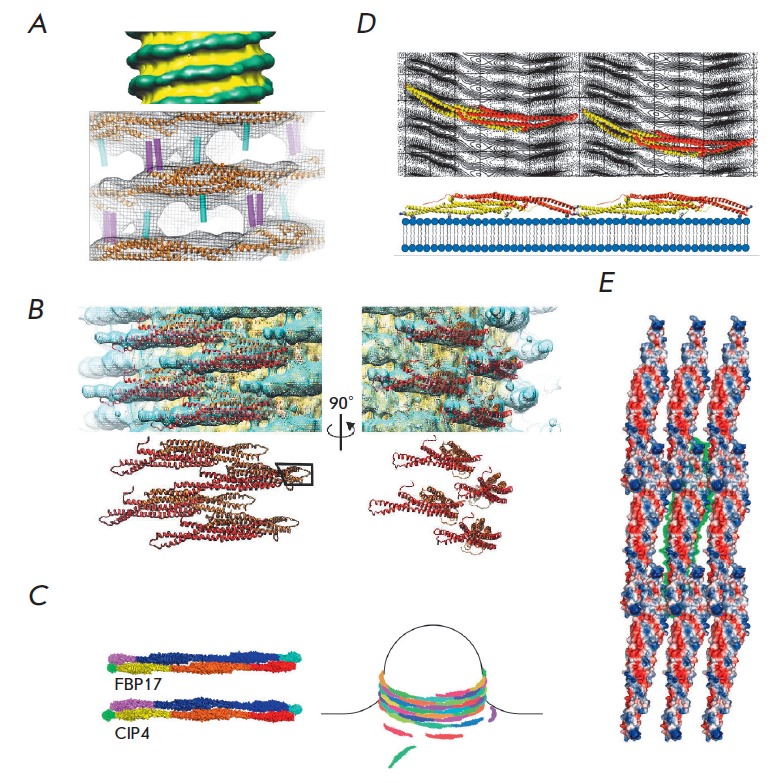
Oligomerization of BAR domains on membranes. *A *– The cryo-electron microscopy model of a 28-nm membrane tubule with endophilin oligomers (top image). The BAR domain (orange) and additional helices (cyan and magenta) are fitted into the electron density [7]. *B *– Oligomerized BAR domains of amphiphysin 2 [72]. *C *– Scheme of BAR domain oligomerization and formation of membrane tubules [16]. *D *– Interactions between dimerized BAR domains of CIP4 [73]. *E *– Interactions between dimerized BAR domains of Pinkbar [45].

Since X-ray diffraction analysis does not provide any idea on how proteins interact with a full-size membrane, reconstructions of the oligomers of BAR domains bound to membrane tubules were obtained by cryo-electron microscopy [7, 72, 73] (*Fig. 3*).

Studies of the arrangement of the F-BAR domains of endophilin on the membrane tubule have demonstrated that they are oriented at a 10° angle with respect to each other [7] (*Fig. 3A*). The large regions of the unoccupied membrane between the neighboring bundles (~50 Å) can be attributed to the need to provide access for GTPases, with which endophilin interacts during endocytosis [74]. When full-length endophilin interacts with a membrane tubule, its SH3 domains are also arranged on the surface as dimers. This has been confirmed by experiments with cross-linking of cysteins inserted into SH3 domains [75]. It was suggested that this spatial organization can be recognized by the GTPase dynamin that carries two neighboring proline-rich sequences [75].

Another oligomeric structure that was studied using cryo-electron microscopy and helical reconstruction was the structure of the BAR domains of the amphiphysin II isoform involved in the organization of T-tubules [72] (*Fig. 3B*). The BAR domains of amphiphysin are more densely packed than those in the endophilin structure and in such a way that one end of the BAR domain is oriented inward into the membrane, while the other one is oriented outwards. Unlike in endophilin, they are stably connected with one another by AHs, which presumably partake in curvature initiation. As a result, the tubules formed by amphiphysin are much more rigid. This agrees well with the biological functions of these proteins: amphiphysin forms stable T-tubules, while endophilin is involved in the formation of the dynamic structures that quickly assemble and disassemble during endocytosis [76].

X-ray crystallography was used to determine the structures of individual F-BAR domains and to propose a scheme of their interaction with membranes. In crystals, F-BAR domains form flat scaffolds where the BAR domains have a lateral orientation. The BAR domains interact with each other through the terminal and lateral regions. When interacting with the membrane, the BAR domains turn so that the curved side carrying the positively charged amino acid residues faces the membrane; the flat scaffold acquires a ring shape, and then it becomes helical and twists around the tubule being formed [16] (*Fig. 3C*). This assumption has been confirmed by cryo-electron microscopy [73] and molecular simulation data [77].

The isolated I-BAR domains can actively form the membrane curvature [33]. However, since this ability is less pronounced in full-length I-BAR proteins [41] due to autoinhibition, they can bind to the already curved membrane. The functions of membrane curvature sensing and generation are not mutually exclusive; hence, it can be assumed that protein behavior depends on its concentration: at low concentrations, they sense the existing membrane curvature and attract other proteins to it, while at high concentrations, they can aggregate into oligomers (*Fig. 3D*) and be actively involved in curvature propagation [78]. On the other hand, the I-BAR domains of the Pinkbar protein form flattened membrane regions instead of curvatures. Accordingly, although containing terminal interactions that are typical of BAR domains, their oligomers are flat (*Fig. 3E*).

**Stabilization of the membrane curvature**

The significance of N-terminal AHs in stabilizing interaction with lipids was established using various methods [15, 79]. In *in vitro *experiments, the absence of AHs made endophilin unable to modify liposomes and form tubules. This has also been demonstrated by molecular dynamics simulation [7]. A more recent study by electron paramagnetic resonance showed that endophilin AHs penetrate into the lipid bilayer by 8–11 Å below the level of phosphate groups and are not in direct contact with each other [80]. A hypothesis has been put forward that the importance of AHs for protein oligomerization can be possibly related to the mutual coordination of lipids. Incorporation of AHs into the top lipid monolayer results in the generation of a positive membrane curvature, due to the asymmetry emerging in the bilayer structure.

The endophilin structure determined by cryo-electron microscopy indicated that the insertions of neighboring (parallel to the long axis of the tubule) dimers do not interact with each other and are oriented towards the membrane. This differentiated them from the arrangement in the crystal structure and in the liposome-bound state. The difference was later attributed to two conformational states: at high protein concentrations sufficient for oligomer formation, the N-BAR domain resides closer to the membrane, thus contributing to a deeper incorporation of AH [80], impeding spontaneous membrane curvature, and stabilizing the membrane tubule. The conformational switch between endophilin states can be associated with the phosphorylation of Ser75: the emergence of a negative charge impedes the incorporation of AH into the membrane and tubule stabilization. The mutations in LRRK2 kinase associated with Parkinson’s disease are known to increase the phosphorylation of Ser75 and cause the disruption of endocytosis in synapses [81].

In addition to attracting partner proteins, the SH3 domain of endophilin regulates the activity of the N-BAR domain. It was demonstrated by a molecular dynamics simulation that the SH3 domain in solution binds to the N-terminal AH due to hydrophobic interactions and the formation of salt bridges between charged residues [8]. The negative electrostatic potential concentrates at the SH3 domain, whereas the positive potential concentrates at the AH. Hence, when a protein approaches the membrane the AH turns towards it, while the SH3 domain turns away from it. On the one hand, the SH3 domain in this autoinhibited form does not interact with other proteins in solution. On the other hand, the protein “searches” for the region in the membrane that would be suitable in terms of electrostatic potential and would have defects in lipid packing, where the AH can be incorporated.

Recently there has been evidence that not all BAR domains exhibit activity in the formation of membrane tubules or membrane invagination. The yeast protein Cdc15p involved in cytokinesis oligomerizes into filaments and does not cause membrane modification [82]. Oligomerization of Cdc15p is needed for a contractile ring to form; however, in this case the protein does not change the membrane’s shape but only helps attract other proteins to it. Lack of tube-forming ability was also demonstrated for six mammalian F-BAR domains. The common function of F-BAR domain proteins possibly consists in the attraction and spatial arrangement of other proteins near the membrane, and only in some cases do they change the membrane shape [83]. The F-BAR domain protein Nervous wreck (Nwk), whose homologues are found in many organisms, from insects to higher vertebrates, is one of such proteins. Two homologues of this protein are involved in membrane rearrangements in mammalian stereocilia and cerebellar neurons [84, 85].

**Nontraditional orientation: The F-BAR domain protein Nervous wreck**

Neuronal growth and the formation of new connections, the processes underlying learning and memory, are controlled by growth factors. Receptors bound to growth factors are moved inside the cell by endocytosis and sent to special cellular compartments, where they can undergo modification or degradation, or interact with other proteins [86]. Determining the mechanisms that control the rate and direction of the flow of receptor-containing endosomes is essential for understanding the signal transduction processes. The neuromuscular junction of *Drosophila melanogaster *is a convenient model for studying synaptic growth regulation, since the muscle area increases more than one hundredfold within four days, which is accompanied by a significant increase in the number of neuronal contacts. Neuronal growth regulation includes both the retrograde signals from the muscle and anterograde signals from the neuron to the muscle cell [87]. Mutations in the proteins regulating endocytosis are known to result in excessive axon branching, since they impede the attenuation of the signal from growth factor receptors [88-90].

**Fig. 4:**
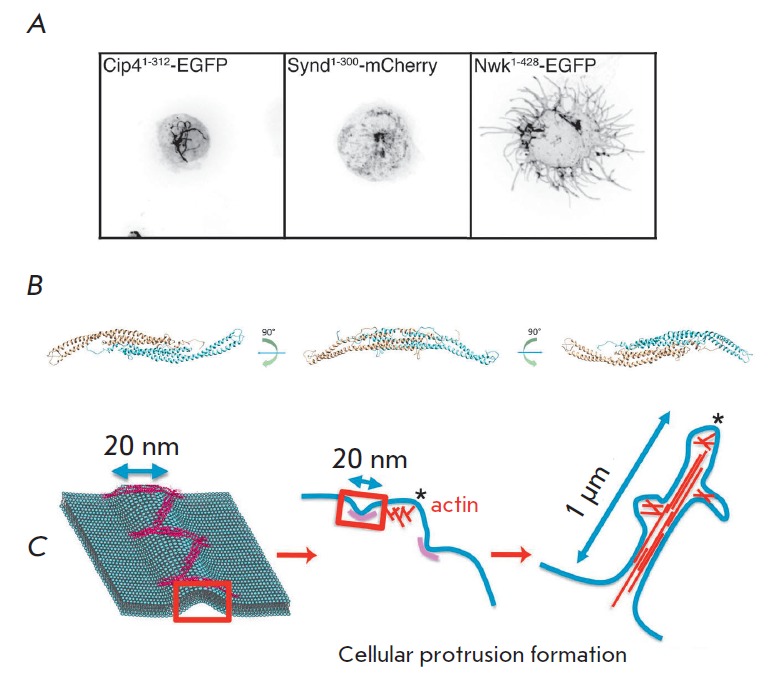
Noncanonical activity of the F-BAR domain of Nwk [92]. *A *– Formation of inner membrane tubules caused by the expression of typical F-BAR domains and formation of cellular protrusions in the case of the F-BAR domain of Nwk. *B *– Model of the F-BAR domain of Nwk (monomers are shown in different colors). *C *– Model of cellular protrusion formation caused by F-BAR domain oligomerization and actin polymerization.

The F-BAR protein Nervous wreck (Nwk) exhibits limited homology to other F-BAR proteins. *In vitro *studies have shown that, unlike other F-BAR proteins, Nwk causes the formation of cellular protrusions rather than invaginations (*Fig. 4A*) [91].

In order to study the interaction between the Nwk F-BAR domain and the membrane, its model was built based on the known crystal structure of the homologous F-BAR domain FCHO_2_, which has a specific S-shape [92] (*Fig. 4B*). Electron microscopy was used to study the ways in which the F-BAR domains of the Cip4 and Nwk proteins were organized on lipids. As expected, the Cip4 F-BAR domains were found to aggregate into linear filaments, while the Nwk F-BAR domains were found to form higher order V-, N-shaped, and zigzag structures [92]. It is important to mention that these structures were not observed in the absence of lipids. The mechanism of interaction between Nwk and membranes was proposed based on these findings. The zigzag structures form a “ridge” on the membrane whose geometry depends on the angle between the dimers and the frequency of the dimers with the concave side facing the membrane. In cells, this ridge can form a ring marking the membrane region that is subsequently transformed into a cellular protrusion by microtubules and actin filaments (*Fig. 4C*) [92].

The end regions of the dimer that are responsible for oligomerization and electrostatic interactions between the membrane and the concave side of F-BAR play a crucial role in this process [92]. Protrusion formation also requires actin filament polymerization; however, the protrusions that have already formed do not respond to treatment with the actin polymerization inhibitor latrunculin B. This fact indicates that actin is required for their formation only [93]. Interestingly, the structure of the emerged protrusions differs from that of filopodia, since the protrusions contain microtubules, along with actin filaments. Treatment with the microtubule depolymerizing agent nocodazole also does not destroy the protrusions.

The functioning of Nwk in neurons is apparently ensured not only by its F-BAR domain, but also by two SH3 domains, each of them binding to certain proteins. Nwk in recycling endosomes interacts with the SNX16 protein belonging to the sorting nexin family, which, in its turn, is bound to the presynaptic growth factor Tkv [94]. The interaction between Nwk and SNX16 reduces the signal from Tkv and is needed to ensure the receptor’s return onto the membrane. Furthermore, Nwk binds to the proteins involved in endocytosis regulation (Dap160, dynamin, and Wsp). Experiments with the mutant SH3a and SH3b domains showed that SH3a binds to dynamin and Wsp, while SH3b is responsible for binding to Dap160 [95]. Wsp activates the Arp2/3 complex, which triggers actin polymerization that is required for endocytosis [96]. However, Nwk activates Wsp in a much weaker fashion than the mammalian SH3 domain proteins (e.g., Nck) activate WASP [97]. The effect can be enhanced by the co-action of Nwk and another activator of Wsp, Cdc42 GTPase. Hence, Nwk interacts with the endocytic machinery through SH3 domains and, together with Cdc42, activates Wsp/Arp2-3-dependent actin polymerization for synaptic growth regulation.

Another important function of Nwk SH3 domains is the regulation of F-BAR activity. The SH3b domain was shown to bind to F-BAR; however, this does not result in a loss of its membrane-binding ability but only increases the amount of the negatively charged lipids needed for binding [10]. Both the F-BAR domain itself and the full-length protein modify giant liposomes; however, their excessively high negative charge prevents membrane deformation [10]. One of the possible explanations is that at lower PI(4,5)P2 concentrations, most F-BAR domains are bound to the membrane by their concave side, which facilitates deformation. On the other hand, it is quite possible that the reason lies in changes in the properties of the membrane itself. The increased PI(4,5)P_2_ concentration in the membrane leads to a rise in the degree of order of lipids, which is characterized by an alignment of hydrocarbon tails, increased bilayer thickness, decreased lateral diffusion coefficient, etc. [98, 99]. The formation of these lipids microdomains may impede membrane deformation or the dynamic migration of proteins that is required for oligomerization [100]. The membrane composition can regulate other BAR domain proteins in a similar way: the increased PI(4,5)P_2_ concentration suppresses the membrane-deforming activity of the F-BAR domain FBP17 in *in vivo *experiments [101]. Binding of SH3 domains to F-BAR was previously believed to result in complete autoinhibition, which can be eliminated either by binding to other proteins [102] or by increasing the negative charge in the membrane [103]. However, the example of Nwk indicates that this mechanism is more complex and requires further research.

## CONCLUSIONS

Deciphering the crystal structures of BAR domains has made it possible to describe the mechanisms of changes in the membrane shape at the molecular level, while *in vitro *studies and electron microscopy have allowed researchers to explain how the schemes of oligomerization of BAR domains result in the formation of various membrane structures. It has been demonstrated how the activity of some BAR domain proteins can be regulated by intra-protein and protein–protein interactions, as well as what the mechanism for achieving a specificity of partner protein recruitment is. However, despite the significant progress in understanding the role of BAR domain proteins in cell activity, many questions still remain to be answered. Taking into account that changes in the expression level and mutations in the genes encoding BAR domain proteins are related to many serious diseases, this field of research is of interest both for biology and medicine.

## Abbreviations

a.a.: amino acid
AH: amphipathic helix
ST: surface tension
PI(4,5)P2: phosphatidylinositol-4,5-bisphosphate
